# Biomechanical Characterization at the Cell Scale: Present and Prospects

**DOI:** 10.3389/fphys.2018.01449

**Published:** 2018-11-15

**Authors:** Francesco Basoli, Sara Maria Giannitelli, Manuele Gori, Pamela Mozetic, Alessandra Bonfanti, Marcella Trombetta, Alberto Rainer

**Affiliations:** ^1^Department of Engineering, Università Campus Bio-Medico di Roma, Rome, Italy; ^2^Center for Translational Medicine, International Clinical Research Center, St. Anne’s University Hospital, Brno, Czechia; ^3^Department of Engineering, University of Cambridge, Cambridge, United Kingdom; ^4^Institute for Photonics and Nanotechnologies, National Research Council, Rome, Italy

**Keywords:** cell mechanics, cell-generated forces, AFM, tweezing methods, MEMS, traction force microscopy, mechanotransduction, mechanobiology

## Abstract

The rapidly growing field of mechanobiology demands for robust and reproducible characterization of cell mechanical properties. Recent achievements in understanding the mechanical regulation of cell fate largely rely on technological platforms capable of probing the mechanical response of living cells and their physico–chemical interaction with the microenvironment. Besides the established family of atomic force microscopy (AFM) based methods, other approaches include optical, magnetic, and acoustic tweezers, as well as sensing substrates that take advantage of biomaterials chemistry and microfabrication techniques. In this review, we introduce the available methods with an emphasis on the most recent advances, and we discuss the challenges associated with their implementation.

## Introduction

Cells are complex biological units that can sense the physico-chemical cues and stimuli from their surrounding environment, and actively respond to them by triggering biomechanical reactions that include cell growth, proliferation, differentiation, motility and even apoptosis (Galbraith and Sheetz, 1998; Huang and Ingber, 1999). Forces generated by cells regulate several biological activities such as cell adhesion, signaling, biochemical routes and metabolic functions, and can be pivotal mechanisms in orchestrating and coordinating the morphogenetic pathways that control tissue and organ development and homeostasis (Mammoto and Ingber, 2010; Maugeri-Saccà and De Maria, 2018; Miroshnikova et al., 2018). In the last decade, several studies focused on the generation, transmission and regulation of signaling pathways and molecular mechanisms within single cells and tissue environments (Geiger and Bershadsky, 2001; Chen et al., 2004; Maloney et al., 2010; Nardone et al., 2017). Advances in cell mechanics have fuelled the development of tools for the mechanical characterization of biological entities at the cellular and subcellular scale (Norman et al., 2008; Zheng and Zhang, 2011). The study of how mechanical properties influence cell behavior in relation to their surrounding microenvironment both in healthy conditions and in disease requires a profound knowledge of extracellular and/or intracellular forces, stiffness values and mechanical stresses from micro- to nano-scales (Butcher et al., 2009; Alcaraz et al., 2018). This knowledge would allow researchers to better understand both the evolution of intracellular architectures and how cells interact with the external environment. This will lead to the investigation of the biomechanical regulation of cell fate in the framework of development, physiology and disease, at the physico-chemical level (Park et al., 2010; Hoffman et al., 2011; Moulding et al., 2012; Sun Y. et al., 2012).

Cells are viscoelastic, and as such they have both an elastic and a time-dependent, viscous behavior. A viscoelastic material typically possesses characteristics such as stress-relaxation, creep, strain-rate sensitivity and hysteresis (Pal, 2014). Stress relaxation occurs when a material is subjected to a fixed strain, and the stress developed by the material is decreasing with time. Creep, on the contrary, is the behavior of a material subjected to constant stress and experiencing a time-dependent elongation. Strain rate sensitivity means that the stress-strain response of a material depends upon the speed at which strain is applied (strain rate). Lastly, hysteresis describes the fact that loading and unloading curves for a viscoelastic material do not follow the same path. The areal difference between the two curves represents the loss of energy due to internal friction in the material.

Recent advances in the development of novel techniques and tools for cell mechanics characterization, and the design of *ad hoc* technological platforms have introduced the possibility to accurately trap and manipulate single cells at the microscale level (Finer et al., 1994; Park et al., 2005; Roth et al., 2013; Guo et al., 2016; and reviewed in Rajagopalan and Saif, 2011; Zheng and Zhang, 2011; Polacheck et al., 2013), for application in many interdisciplinary areas of research, such as biophysics, biomedicine, tissue engineering, and materials science.

Here, we will summarize the latest advances in the research area of cell biomechanics, and we will focus on the modern technological approaches and mechanical testing systems developed in the last decade by combining theoretical, experimental, and numerical models, for pursuing a realistic description of cell mechanical behavior. First, we will introduce the established techniques and available tools, highlighting the differences between active and passive stimulation methods. We will provide a brief description of atomic force microscopy (AFM) and AFM-derived methods, and then we will explore thoroughly the tweezing methods, including optical, magnetic and acoustic tweezers. Also, we will outline the role of microengineered platforms, such as Micro-Electro-Mechanical Systems, micro/nanopillars, microfluidic devices, and hydrogel stretching methods (highlighting the underlying technology and mathematical modeling) for cellular force measurements. Finally, we will critically discuss the future outlooks of such technological tools and the challenges that still need to be addressed to understand the structural and mechanical complexity of living tissues.

## Classification

Measuring forces at the cell–extracellular matrix (ECM) interface is a critical aspect for fully understanding cell–ECM interactions and how the ECM regulates cellular function. This has boosted the development of technological platforms achieving force measurements at the cellular and subcellular scale.

It is possible to divide these technologies in two broad categories: (i) active stimulation methods, which measure cell response to mechanical force application, and (ii) passive stimulation methods, which can only sense mechanical forces generated by cells without applying any external force.

Mechanical cell responses to external inputs have largely been studied using active single-cell manipulation approaches, such as:

• Atomic force microscopy (AFM) (Lam et al., 2011): AFM relies on microcantilevers to induce a deformation in the cell. From the deflection of the cantilever, it is possible to measure local mechanical properties and to generate maps across the cell surface.
• Tweezing methods, which encompass three main techniques.
  – Optical tweezers (OTs) (Galbraith et al., 2002): OTs rely on a laser beam to create a potential well for trapping small objects within a defined region. Optical tweezers can be used to micromanipulate cells as well as intracellular components (i.e., organelles) and quantitatively measure the binding force of a single cell to diverse types of ECM substrates (Guck et al., 2001; Wang et al., 2005), or to evaluate physical interactions between subcellular structures (Sparkes et al., 2018)
  – Magnetic tweezers (MTs) (Hu et al., 2004): these devices rely on the use of magnetic microbeads. Magnetic fields are produced either by movable permanent magnets or by electromagnets (Ziemann et al., 1994).
  – Acoustic tweezers (ATs) (Guo et al., 2015): ATs can manipulate biological samples using sound waves with low intensity power and low impact on cell viability, and without the need for any invasive contact, tagging, or biochemical labeling.

In the passive methods, the main goal is the evaluation of cell-generated forces using flexible substrates:

• Microengineered platforms: these are microfabricated platforms, including both silicon-based devices (micro-electro-mechanical systems, MEMS) produced through integrated circuit manufacturing processes, as well as elastomeric (i.e., polydimethylsiloxane, PDMS) devices produced through replica molding (Tan et al., 2003; Kim et al., 2009).
• Traction Force Microscopy (TFM): TFM exploits elastic substrates with known mechanical properties and fluorescence/confocal microscopy. In its original version, cells were cultured on flexible silicone sheets with different compliance. During cell action, silicone patterns wrinkled and this could be visualized under a light microscope (Harris et al., 1980). An evolution of this method implies the use of flexible sheets with embedded beads. Positions of the beads are tracked during the experiments and cell-generated foces are derived from the analysis of bead displacement field (Lee et al., 1994).

A summary of the available techniques with a brief description of their advantages and disadvantages, their range of detection, and a simple sketch is reported in Table 1.

**Table 1:** Summary of the most relevant techniques for cell mechanical characterization.

Mode	Technique		Sketch	Typ. force range	Strengths/limitations	References
ACTIVE	AFM		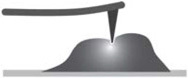 Adapted from: Liu et al. (2012)	5 pN ÷ 10 nN	✓ Wide force range –well established technique × Direct contact with the specimen otherwise tip must be labeled	Lam et al., 2011
	TWEEZING	OPTICAL	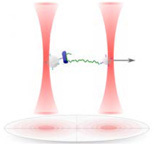 Adapted from: Håti et al. (2015)	0.1 ÷ 100 pN	✓ High sensitivity ✓ Wide force range × Heating issues	Guck et al., 2001; Galbraith et al., 2002; Wang et al., 2005
		MAGNETIC	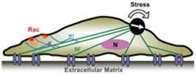 Adapted from Poh et al. (2009)	0.01 ÷ 100 pN	✓ High sensitivity ✓ Wide force range × Custom equipment – little standardization	Wang et al., 1993; Ziemann et al., 1994; Hu et al., 2004
		ACOUSTIC	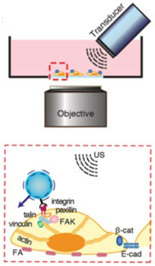 Adapted from: Topal et al. (2018)	0.1 ÷ 30 nN	✓ Highly cytocompatible × Fewer force and stress application modalities	Ding et al., 2013; Guo et al., 2015; Li et al., 2015
PASSIVE	MICRO-ENGINEERED PLATFORMS		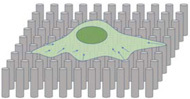	10^−12^ ÷ 10^−3^ N	✓ Different designs translate into extremely wide force range ✓ Stability, scalability of manufacturing process ✓ Sophisticated microfabrication procedures/facilities	Yang and Saif, 2005; Polacheck and Chen, 2016
	TRACTION FORCE MICROSCOPY		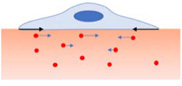	2 ÷ 120 nN	✓ Widespread (needs standard lab equipment) ✓ Easily coupled with microscopy equipment (i.e., fluorescence/confocal) ✓ 2D/3D measurements × Computationally demanding × Non-linear behavior of ECM-mimicking hydrogels	Polacheck and Chen, 2016

*For each technique, the force range and a brief description of the main strengths and limitations are reported.*

## Atomic Force Microscopy (AFM)

Among the available techniques for measuring the mechanical properties of biological tissues, AFM has been extensively used over the years thanks to its capability to cover, with nanometer resolution, the pathophysiological range of stiffness values of tissue samples, while probing local cell-ECM mechanical interactions (Dufrêne and Pelling, 2013).

AFM is a very powerful technique that allows cell biologists to probe the morphology of living cells, mechanical and adhesive properties of single biomolecules, as well as to quantify and to spatially map cell mechanics and physical properties. AFM can operate under a wide variety of physiological conditions: biological samples can be imaged in fluid environments, and live-monitored in real-time. Moreover, AFM can be combined with many optical microscopy techniques (e.g., correlated fluorescence-AFM studies) (Kodera et al., 2010; El-Kirat-Chatel and Dufrêne, 2012; Martinez-Martin et al., 2012) to simultaneously visualize single cells to extract additional information. An in-depth description of AFM functioning principles is not in the scope of the present review. For a more detailed description of the AFM, the reader may refer to Dufrêne and Pelling (2013) and Kasas et al. (2018).

Designed to study surface morphology, AFM has been used for many years as a tool for high-resolution imaging of surfaces. AFM uses a very sharp silicon micro-fabricated tip fixed at the end of a cantilever beam to scan the surface. After positioning the tip a few nm from the scanned surface, this is subjected to a short–range interaction with the sample during which the cantilever deflects. Deformations are recorded measuring the angular deflection of a laser beam aligned to the cantilever end on a multi-segment photodiode. Thus, while the tip raster scans the sample in the X-Y directions with very precise piezoelectric scanners, a detailed image of the 3-D topography of the sample is generated (Kasas et al., 2018).

Besides imaging, AFM force spectroscopy has been also used to apply mechanical forces to biological systems over scales ranging from cells to single molecules to study their mechanical response (Zemła et al., 2018) and measure their mechanical and adhesive properties.

An early approach to measure nano-mechanical properties of biological samples consisted in the analysis of specimen height profile when subjected to multiple scans at different force levels through the AFM tips, estimating the elastic modulus by the force-deformation relationship (Kis et al., 2002).

Further development involved the use of the tip-cantilever system as an active scanning probe. In the “pushing experiments” the AFM tip is indented against the sample while recording its response through a force/distance curve, or force/indentation curve, which represents the deformation of the cantilever given a prescribed force or the required force to push the tip to a definite depth into the sample (Zemła et al., 2018).

The recorded curve can be used to calculate the sample stiffness by algorithms that take into account several parameters, such as the geometry of the tip and the depth of indentation (Harris and Charras, 2011; Dufrêne and Pelling, 2013). Alcaraz et al. (2018) presented a very exhaustive methodology to select the right tip geometry (from the four-sided pyramidal or the spherical commercial tips to the cylindrical tips made by milling a commercial pyramidal tip by mean of a focused ion beam) considering the topology of the sample to be studied and the goal of the study.

Several studies focused on the computational modeling of the force-versus-indentation curve, where different contact models between cell and tip have been proposed in order to properly account for different geometries: Hertz model (Hertz, 1896), which describes the elastic deformation of two spheres, Sneddon model (Sneddon, 1965) which describes the elastic deformation of conical or paraboloidal tips on a flat sample, JKR model, which also considers adhesive forces, Tatara (1989) model, case of a spherical sample compressed between two parallel plates, Multiscale Decomposition Analysis (Digiuni et al., 2015), which quantifies the non-linear mechanical response of the cantilever above cell wall indentation, and the Multi-Regime (Bonilla et al., 2015), which considers cells as a multi-spring system. Lately, to overcome a common drawback to these methods (i.e., sample, tip and substrate are assumed to be homogenous bodies with well-defined geometries, a rough approximation of real conditions), the use of finite element modeling (FEM) is emerging as a possible solution in order to better consider the local effects of the tip–sample interactions (Kasas et al., 2017) on the recorded measurements.

A remarkable example of the importance of adopting the right computational model in AFM data analysis is reported by Mercadé-Prieto et al. (2013). They showed that the stiffness values measured by AFM indentation on *Saccharomyces cerevisiae* cell wall were two orders of magnitude lower than those obtained by micromanipulation studies. The authors ascribed such discrepancies to the use of mathematical models that are inappropriate to fit the experimental data. In fact, the classical Hertz-Sneddon model, based on the assumption that the whole cell is a homogeneous material, does not hold for tissues with a complex hierarchical structure. The problem was solved by implementing a new FEM-based model, which considered the yeast cell wall as made of a soft external layer and a stiffer inner layer.

Another relevant AFM application is represented by the so called “pulling experiments”. In this case, the tip of the AFM is used to pull the cell instead of applying a compressive force on it. This technique allows to localize cell surface molecules and to study their elasticity and adhesion (Hinterdorfer and Dufrêne, 2006; Puchner and Gaub, 2009).

The AFM tip often needs to be functionalized with specific moieties or biomolecules that specifically interact with the surface of the cell. Once the probe is brought in contact with the cell surface and a molecular interaction occurs between the tip and the probed molecule (contact time), the cantilever position is perturbed. The tip will firstly bend downwards while being retained by the interacting molecules. Ultimately, the retracting force possessed by the cantilever beam will lead to the detachment of the modified AFM tip from the investigated surface. Therefore, it is possible to measure the force applied on the cantilever beam by the interacting molecules and to draw a retraction curve that contains characteristic “rupture events” that represent the required force to break a single molecule interaction. Moreover, by imposing different stretching speeds, it is possible to evaluate the potential energy parameters of the molecular interactions, leading to an estimation of the binding strength of a given receptor (Karácsony and Akhremitchev, 2011; Dufrêne and Pelling, 2013).

An interesting approach, exploiting AFM in a passive mode, is represented by the work of Liu et al. (2012), who developed a technique to study the contractile force of cardiomyocytes. They first brought the AFM cantilever to gently touch the living, beating cells, then locked the z-piezo and let the contraction forces of the cell deflect the cantilever. This method allowed to quantitatively measure several parameters, including elastic modulus, contractile force, beat rate and beat duration.

The understanding of how nanomechanical forces induce signaling, and how this is transmitted through the cellular architecture was achieved by the combination of AFM and optical techniques.

AFM mechanical measurements made while scanning the cells with different optical microscopy procedures (Lehenkari et al., 2000; Haupt et al., 2006), such as fluorescence microscopy or laser scanning confocal microscopy, led to many important observations on the origins of mechano-sensitivity and the processes of mechano-transduction in living cells. Several groups have independently demonstrated how cells are extremely sensitive to small local forces by using fluorescent fusion proteins coupled with live cell dyes. For example, the local indentation of living cells with an AFM tip with forces in the order of 10^−9^ N, influences their behavior making them generate an inner response by signaling and structural events (e.g., calcium release, membrane blebbing, cytoskeletal deformation, and organelle rearrangement) (Charras and Horton, 2002; Silberberg et al., 2008; Veraitch et al., 2011; Guolla et al., 2012). AFM records force-distance curves by plotting the force acting on the probe as a function of the probe-sample separation distance. Nanomechanical and nanoadhesive properties of the living material can be extracted from the experimental data by using the available physical models (Formosa-Dague et al., 2018).

Many studies have been conducted using AFM as a probe for the understanding of cell behavior under external mechanical stress. Those studies showed the wide range (from as low as 0.02 kPa up to 400 kPa) of the elastic modulus of living cells, as it has been extensively reviewed and accurately tabulated by Kuznetsova et al. (2007).

In the standard indentation method, cells need to be firmly attached to the substrate. This may raise problems for non-adherent cells in suspension. Many methods have been presented to overcome this issue, and have already been described in the literature (Kuznetsova et al., 2007). However, effects on the cell elastic modulus caused by the immobilization (Dulińska et al., 2006) (e.g., poly-L-lysine solution for native erythrocytes attachment to glass surface, Dulińska et al., 2006) should be expected and they should be taken into account during the estimation of the elastic modulus to avoid under or over estimation (Rosenbluth et al., 2006). Moreover, since cells are not homogenous, heterogeneous mechanical properties can be expected at different positions within the cell. For example, Mathur et al. (2000) reported high variation in the elastic modulus for human umbilical vein endothelial cells (HUVEC) measured over the nucleus, in its proximity, or near the edge of the cell body, with the nuclear area being stiffer than the rest of the cell body. Similarly, variability of local mechanical properties was reported for many other cell types, including bovine pulmonary artery endothelial cells (Costa and Yin, 1999), cardiomyocytes (Shroff et al., 1995), etc. Cell thickness is also a factor to be taken into account, as it can affect the measurement of the elastic modulus (Mahaffy et al., 2004).

Even though the interpretation of AFM data is not straightforward, this technique has the advantage to be coupled with microscopic observation, for the convenient analysis of the mechanisms of cell function through a deeper knowledge of the cell behavior under stress (Kuznetsova et al., 2007). For example, Pesen and Hoh (2005) perturbed the assembly of cytoskeleton components using drugs. By combining AFM analysis with confocal fluorescence microscopy (CFM), they were able to study the subcellular organization of different cytoskeletal components, clearly showing that the elastic response of cells is mostly due to the network of actin filaments. With the combined AFM-CFM approach, they also characterized the local micromechanical architecture of the cell cortex in bovine pulmonary artery endothelial cells.

More recently, Fallqvist et al. (2016) used a drug that disrupts the cellular actin network (Latrunculin B) to study how the actin cytoskeleton influences the mechanical properties of fibroblasts. They confirmed that the disruption of the actin network reduced cellular stiffness, while increasing the relaxation rate of the cytoplasm. They hypothesized that the actin network is responsible for both the viscous properties of the cell and its stiffness. Similar experiments, based on the disruption of the cytoskeleton using different molecules (e.g., cytochalasin D and nocodazole for actin microfilaments and microtubules, respectively) in order to evaluate their connection with cell mechanical properties, were extended to primary chondrocytes, endothelial cells, fibroblasts, hepatocellular carcinoma cells, and fibrosarcoma cells (Grady et al., 2016).

AFM allowed scientists to take another step forward in the understanding of the relationship between mechanical properties and cell behavior during the mechanisms of cell differentiation and aging. Reorganization of cytoskeleton and modifications in mechanical properties seem to be correlated with the cell cycle stages (Collinsworth et al., 2002; Zhang et al., 2004). AFM studies conducted on HUVEC (Sato et al., 2004) and human epithelial cells (Berdyyeva et al., 2005) correlate cell elasticity and culture period. The obtained values of stress over time show an increase in *in vitro* cell rigidity during aging. Lieber et al. (2004) also used AFM nanoindentation on isolated cardiomyocytes from young and old male hybrid rats (Fischer 344 Brown Norway F1), discovering a correlation between the increase in their apparent elastic modulus and aging.

Understanding the effects of diseases on cell mechanical properties also represents a major application of AFM. The direct link between structure and functions is the reason for the increasing number of studies that aim to correlate the change of the mechanical properties with pathological conditions involving numerous diseases and biological structures. These aspects have extensively been reviewed (Morton and Baker, 2014; Gautier et al., 2015; Dinu et al., 2016; Rianna and Radmacher, 2016) and will be summarized next. A notable example of the AFM application in this context comes from the work of Sun N. et al. (2012). As a model for investigating dilated cardiomyopathy (DCM), the authors generated cardiomyocytes (CMs) derived from induced pluripotent stem cells (iPSCs) from patients with a familial point mutation in the gene encoding cardiac troponin T. They measured the contractile force of such iPSC-CMs through AFM using a silicon nitride cantilever tip with a cellular indentation of around 100–200 nm, and applying a 100 pN contraction force to cells. This technique allowed them to detect much weaker single-cell contraction forces in the DCM iPSC-CMs compared to control iPSC-CMs from healthy individuals of the same family cohort.

Another remarkable example of using AFM to perform force mapping measurements between normal and defective cells was recently given by Nardone et al. (2017). In this work, the authors studied the interplay between the activity of the Hippo pathway effector Yes-Associated Protein (YAP) and formation of focal adhesions (FAs, sub-cellular protein complexes that act as linkages between integrin-ECM connection and the cytoskeleton) in physiological and simil-pathological conditions. They compared genetically modified YAP-deficient adipose tissue-derived mesenchymal stem cells (AD-MSCs) grown onto different substrates with normal AD-MSCs via force mapping AFM measurements, thus providing novel insights into the mechanism of YAP mechanosensing activity in regulating the assembly of FAs.

Considering their important role in numerous physiological and pathological processes (atherosclerosis, blood pressure regulation, etc.), mechanical alterations of endothelial cells due to pathological events have been extensively studied (Szymonski et al., 2015). For example, AFM indentation measurements on *ex vivo* specimens of abdominal and common iliac aorta from cholesterol-fed rabbits were performed with the aim to assess the mechanical properties of endothelial cells as a function of the biological site and of disease progression (Hayashi and Higaki, 2017). Further, it was demonstrated that cholesterol greatly increased both stiffness and viscosity of human umbilical cord vein endothelial cells (Yan et al., 2017)

Dulińska et al. (2006) investigated the changes in the elastic modulus of erythrocytes from patients with different types of anemias, concluding that the elastic properties of pathological erythrocytes are two to three times higher than normal cells. Besides anemia, erythrocytes were shown to modify their mechanical properties also in other pathologies (e.g., Parkinson’s disease, Alzheimer disease, etc.), as summarized in previous reviews (Mukherjee et al., 2015).

In recent years, the discovery that the mechanical properties of single cells are closely related to cancer development has significantly increased the number of publications in this area. Many of these studies are based on the possibility of using AFM as an early cancer detection instrument, by exploiting the nano-mechanical modifications induced by cancer (Kasas et al., 2018). AFM has been used in many studies on different cancer typologies (e.g., bladder, Abidine et al., 2015; cervix, Zhao et al., 2015; oral mucosa, Park et al., 2016; bone, Wang et al., 2016; prostate, Efremov et al., 2015; lung, Han et al., 2016; brain, Ciasca et al., 2016). All these studies agree that it is possible to distinguish cancer cells from their healthy counterparts by analyzing their mechanical properties and correlating them with other relevant cell features, such as morphology, migration potential and invasiveness (Lekka, 2016). We report here a few examples of the ongoing research on one of the most common type of cancer in woman, that is breast cancer.

Plodinec et al. (2012) demonstrated the strong link between cancer progression and a substantial softening of tumor epithelial cells compared with normal mammary tissue using AFM high-resolution stiffness mapping. They suggested a direct connection between metastatic potential of cancer cells and cell softening in the primary tumor. Ansardamavandi et al. (2016) used AFM to analyze *ex vivo* bioptical specimens of breast tissue, showing a general stiffness modification in cellular and non-cellular regions associated to cancer development. In particular, they reported softening of the cellular regions of cancerous tissues compared to their healthy counterparts, while the fibrous regions slightly stiffened. Another study by Coceano et al. (2016), based on the comparison of the elastic modulus of different human breast cancer cell lines, showed that cell aggressiveness (and hence infiltration potential) correlated with a reduction in cell stiffness. This research showed that AFM indentation technique is potentially able to probe human breast biopsies at the tissue level, representing a possible marker for cancer diagnosis in future.

As a last remark of this section, it has to be pointed out that AFM analysis is generally restricted to the outer surface of cell membranes and it is not able to directly investigate intracellular structures, as the cantilever cannot scan the inside of a cell membrane. To overcome this limitation, Usukura et al. (2012, 2016) have applied an “unroofing” methodology, consisting in the breakage of the cellular membrane and the removal of cytoplasmic-soluble component, to allow the AFM probe directly access cytoskeleton and organelles.

## Tweezing Methods

Mechanical forces generated by cells, along with those developed by the surrounding environment and externally applied to cells, are involved in the regulation of the physiological functions of bio-molecules. Besides leading the required actions for tissue development and homeostasis (i.e., stretching, bending, repositioning, and alignment), these forces also enable and regulate cell functions such as activation of signaling pathways, transcription, cellular differentiation and proliferation (Polacheck and Chen, 2016). Such forces, acting at the molecular level, range from a few piconewtons to several nanonewtons. To investigate those mechanical properties of biomolecules and their interactions, many different devices and methodologies have been developed. Among them, tweezers have undergone a significant development in the last three decades, allowing the manipulation of individual molecules within cells with extraordinary precision. These powerful single-molecule tools include: (*i*) optical tweezers for high precision measurements of forces throug optical micromanipulation, down to the single-molecule level (Hénon et al., 1999)*;* (*ii*) magnetic tweezers for the simultaneous manipulation and recording in real time of forces using tethered magnetic beads; (*iii*) acoustic tweezers for manipulating cells in three dimensions using sound waves.

### Optical Tweezers (OTs)

Laser trapping, better known as OTs, and invented by Ashkin et al. (1986), became one of the most widely used single molecule tools in biology. In fact, OTs are especially suited to manipulate mesoscopic systems, which are characterized by forces ranging from femtoNewtons to nanoNewtons, length scales ranging from tens of nanometers to hundreds of micrometers, and time scales ranging upward from one microsecond (Grier, 2003; Fazal and Block, 2011). These ranges cover those experienced by biological molecules in their native environment (i.e., many of the inter- and intracellular processes). The working mechanism of OTs is based on focusing a laser beam, introduced through a high numerical aperture objective, onto a dielectric micro-particle, in such a way that the interaction with the laser light stably traps the particle close to the beam focus. Usually polystyrene or silica microspheres are bounded to molecules of interest and, upon being trapped by the OTs, used as handles for the manipulation of cells. Forces arising from the radiation pressure of a highly focused light beam are responsible for the dielectric particles entrapment (Ashkin et al., 1987), where the dominant component of the force is along the gradient of the electric field and pushes the dielectric particles toward the center of the focused beam. In a small region around the center, the trap behaves like a linear Hooke’s spring. The value of the trap elastic constant is determined by calibration techniques, normally by thermal fluctuations of the position of the trapped particle (Lisica and Grill, 2017). Therefore, once a particle has been trapped, it is enough to change the position of the focus to move it, using the laser beam in the same way of a pair of tweezers. Thus, one of the main advantages of OTs over other techniques, such as MTs (described below), is represented by the possibility to modify the trap position in all three dimensions at high frequencies by moving the laser beam. For example, this is required to probe multiple conformational states of microscopic particles, such as proteins.

Many OT configurations and geometries exist to fulfill the diverse biological applications that have been studied in the last three decades. They can be divided in two large groups: static configurations (i.e., single bead, two-bead, three-bead), and dynamic configurations (i.e., force-clamp, position-clamp, Dynamic Force Spectroscopy). The reader will find a deeper and more detailed description of such OT configurations and geometries in the reviews and articles that have been written on this subject (Capitanio and Pavone, 2013).

In the study of mechanical properties, the use of microbeads as handles (or grips) to probe force is necessary to overcome the fact that most cells, due to their size, shape and adherent properties, are not conducive to direct optical tweezing. While some cell types – e.g., red blood cells (RBCs), yeast cells and spermatozoa – are easily tweezed (Dholakia and Reece, 2006), the majority cannot, demanding for alternative models (Zhang and Liu, 2008). For example, thermal and hydrodynamic effects on the biomechanical properties of biological cell membrane in physiological flow were studied by OTs using unilamellar vesicles (Foo et al., 2003, 2004), which are widely accepted as a model for studies over cell mechanical properties (Ichikawa and Yoshikawa, 2001).

Concerning RBCs, since it was shown that their mechanical properties change following structural or molecular alterations induced by different kind of diseases (e.g., gastrointestinal tumor and malaria) (Suresh et al., 2005), studies of their biomechanical properties have been performed by OTs using silica beads bound to the membrane, in either single-trap (Lim et al., 2004) or dual-trap (Hénon et al., 1999) configuration. Among the technical improvements for RBC studies, it is worth reporting the following two techniques: (*i*) a three-laser trap setup that has been used to initially stretch RBCs in different directions and then, by simultaneously removing the three OTs, to study the recovery and the cell relaxation time (i.e., time taken by a deformed cell to go back to the undeformed state), which differentiates the cell age (Bronkhorst et al., 1995); (*ii*) the use of a focused evanescent wave illumination technique, which goes under the name of single beam near-field laser trapping, which allows stretching, rotating and folding RBCs (Gu et al., 2007).

A significant advancement in analysis throughput using optical trapping has been reported by Guck et al. (2005) who used two counter-propagating divergent beams to stably trap and deform cells flowing in a microfluidic channel, thereby achieving flow-cytometric measurement of single cell viscoelasticity.

OTs have also been used to study cell membranes and subcellular organelles (Wei et al., 2008; Yalcin et al., 2009). In this case, it is typical to use micron-sized beads anchored to the subcellular structure of interest. OTs have been used to stretch chondrocytes to measure the bead/membrane tether formation force, uncovering that the process of chondrocyte adhesion is directly related to the culture time (Huang et al., 2003). The different tether length of fibroblasts and human mesenchymal stem cells (hMSCs) was also measured, demonstrating that membrane mechanics dramatically affects MSC differentiation (Titushkin and Cho, 2006). Among the other applications of OTs, it is worth reporting their use in different human cell studies, such as: holographic OT techniques to investigate hyaluronan-mediated adhesion processes of chondrocytes (Curtis and Spatz, 2004) and OTs experiments for elucidating the short-term binding of fibroblasts to fibronectin-coated glass (Thoumine et al., 2000), the mechanics of cellular adhesion to artificial artery polymer templates (Knöner et al., 2006), and the interaction forces between human bone cells and implant surfaces (Andersson et al., 2007).

Besides allowing a dynamic analysis of the mechanical properties of cells (Huang et al., 2003), OTs can be also used in the study of tissues (López-Quesada et al., 2014) without alterations to the embryonic development. When using lasers on biological samples, there is always a big concern regarding photodamage. Considering that biological samples are almost transparent to the near-infrared wavelengths that are normally employed to trap particles by laser traps (Neuman et al., 1999), this problem is minimized. Thus, this compatibility with cellular specimens allows the use of OTs in living cells (Monachino et al., 2017). In this field, it is worth to highlight the papers by Nan et al. (2008) and Sims and Xie (2009), in which OTs were developed and used for sub-millisecond tracking of organelles cargoed by the molecular motors kinesins and dyneins. Hendricks et al. (2012) applied OTs to study bi-directional transport of phagocytosed latex beads (LBCs) along microtubules of living mammalian macrophages, suggesting that bidirectional transport of LBCs is driven by opposing teams of stably bound motors that operate near force balance. Thus, OTs offer a wide set of methodologies that make them suitable for trapping and potentially measuring fluid forces also *in vivo*. A breakthrough in that direction was made by Zhong et al. (2013) who trapped RBCs using OTs in the capillary vessels of mouse ears. Clearly, this approach shows limitations when the events of interest are located deep in tissues. Other examples of studies in tissues are given by the recent work of Bambardekar et al. (2015), who accurately measured the tension forces between adjacent cells of a Drosophila embryo by deforming cell junctions with oscillating OTs, and by Johansen et al. (2016), who demonstrated the potential of optical tweezing in a living zebrafish (ZF) embryo exploiting its relative optical transparency. One of the major obstacles for all the *in vivo* studies is the calibration of the optical trap, since the heterogeneity of both cell size and refractive index limits the use of OTs in realistic *in vivo* contexts. Recently, the feasibility of *in situ* calibration of the optical trap stiffness (k) and the position detection sensitivity (1/β) has been demonstrated *in vivo* in the yolk sac of living ZF. Moreover, the study provided measures of the viscoelastic properties of ZF embryo yolk over an order of magnitude of stress-strain amplitude (Staunton et al., 2017). Harlepp et al. (2017) demonstrated the possibility to use OTs for measuring flow profiles and drag forces imposed to trapped RBCs of living ZF embryos.

### Magnetic Tweezers (MTs)

MTs arose as one of the most widely used and powerful tools for the analysis of molecular forces and for the micromanipulation of cells, mainly thanks to their capability of applying torque (Oberstrass et al., 2012). The possibility to be integrated with a range of powerful light microscopy imaging modes, as well as their high force, spatial, and temporal sensitivity (Gosse and Croquette, 2002; Kilinc and Lee, 2014) have greatly widened MTs applications.

The development of MTs is relatively recent and allows the application and measurement of forces ranging from pico- to nanoNewtons using magnetic micro-beads, under a generated magnetic field gradient. Therefore, MTs are mainly used in biological applications for: single molecule force measurements (SMFM) or extra-cellular and intra-cellular (magnetic beads are located outside and inside the cell, respectively) micromanipulation. Moreover, similarly to other biophysical methods, MTs have a very low interference with the specimen, which is very important in research involving mechanotransduction (De Vlaminck and Dekker, 2012; Oddershede, 2012; Monachino et al., 2017).

A typical MTs setup is composed by a tracking system, which is usually made of an optical microscope and various magnetic elements that can be made either by permanent magnets or electromagnets (Xin et al., 2017).

All the components are normally chosen and assembled for the specific force to be applied to the system. It is possible to control the magnetic force by changing the size and shape of the magnet, by changing its magnetization orientation, or by varying the distance between the magnets and the magnetic beads. MTs allow the application of stretching/pulling forces, which are perpendicular to the biological sample substrate, or twisting forces parallel to the biological sample substrate (Shang and Lee, 2007; Kilinc et al., 2012; Tabdili et al., 2012). These operations are even easier by using electromagnets, i.e., generating the field by electrical current passing through a coil. Likewise, it is possible to precisely control the magnetic field and to easily switch its magnitude and direction and the number of poles.

Most magnetic particles used as force transducer in MTs studies are superparamagnetic (SPM) or weakly ferromagnetic. SPM particles are mainly used for their relatively high susceptibility and zero residual magnetization (O’Mahony et al., 2013), while ferromagnetic nanoparticles, thanks to their high saturation magnetization, are normally chosen where there is a weak external magnetic field and the particle size is limited.

Despite a great share of research on MTs concentrates on the development of electromagnets and system design, yet the experiments are typically based on commercial magnetic beads; thus, the possibility to widen their properties and characteristics by achieving different shapes, sizes and element compositions is pivotal for enhancing MTs performances (Zhang and Wang, 2012; Tavacoli et al., 2013). Nowadays, many companies sell iron oxide SPM beads with diameters ranging from 0.1 to 100 μm and with a wide selection of chemically modified or biologically functionalized surfaces. As an example, nanorods, thanks to the possibility of combining multiple metallic elements (Zhang et al., 2011; Lin et al., 2012) or alloys (Zhang and Wang, 2012) into a single structure, have been successfully used to enhance properties for target applications (e.g., in torque measurements the need to have small particles with high torque sensitivity and small stretching force) or to introduce multiple functionalities, such as magneto-optically active materials (Zhang et al., 2011).

In the near future, novel MTs probes made of magnetic beads with enhanced properties, such as programmable shape changes (Tavacoli et al., 2013) or collapsing under the exposure to weak magnetic fields (Fuhrer et al., 2013), could further widen the application fields of this technique.

MTs were subject to many updates and enhancements through the years, and some of the early limitations (i.e., the extreme difficulty to measure torques in the range of 10 pN⋅nm, typical of DNA, the impossibility to decouple twisting torque from stretching force, and the necessity to apply an external torque in order to study the natural torque of a molecule), were overcome by alternative approaches that have been widely described in many reviews (Kilinc and Lee, 2014). For example, approaches like “soft MTs” (Mosconi et al., 2011), “rotor bead tracking” (Oberstrass et al., 2012), “freely orbiting MTs” (Lipfert et al., 2011) and “magnetic torque tweezers” (Lipfert et al., 2010) were developed to decouple torque from stretching force. Moreover, the raise of a series of instruments called “electromagnetic torque tweezers” sensible to stretching forces from 10 fN to 10 pN and with torsional stiffness ranging from zero to 1000 pN⋅nm/rad (Janssen et al., 2012), creates a category of instruments with broad ranges of force and torque. Other approaches also make the most of the simple and flexible MTs design by combining it with other force probing techniques (e.g., OTs, TFM, and many others), building a huge variety of instrumental setups tailored to different applications. For a more detailed insight on the technical aspects of MTs we suggest to refer to Kilinc and Lee (2014) and Tanase et al. (2007).

MTs equipment is easy to assemble and cheap. However, MTs are not widely commercialized and research laboratories possess their own highly customized tools, thus they are not standardized. This aspect is of particular importance considering that the use of electromagnets may affect the behavior of biomolecules by hysteresis of the magnetic field and heat generation around the sample (Rocha, 2015).

In the near future, the integration with other biophysical measurements, the development and fabrication of new classes of multi-functional magnetic particles with enhanced magnetic properties, or different programmable geometries, could expand the potential uses of MTs pushing forward the current boundaries of the methodology.

MTs have been used for analyzing local viscoelastic properties of the cytoplasm (Bausch et al., 1999), for testing different membrane structures with distinct characteristics as in the case of intracellular organelles, for probing the molecular basis of cell mechanics (including the linkage mechanisms of transmembrane integrins to different component of the cytoskeleton), and for studying the chromatin structure, function, and the detailed nuclear architecture.

MTs have been used in several studies to explore how cells respond to mechanical forces. Wang et al. (1993) applied a twisting force to integrins on the surface of endothelial cells, using arginine-glycine-aspartic acid (RGD) peptide-coated magnetic beads, observing the actin cytoskeleton dependent stiffening response. Glogauer et al. (1995, 1997) placed permanent magnets over cell cultures in order to vertically pull collagen-coated magnetic beads attached to the cell surface. Using this method, they were able to perform both single cell analysis, for example measuring a modification of the intracellular calcium content in response to force, and bulk biochemical measurements on large populations of cells, that allowed them to show an increase of protein tyrosine phosphorylation in response to force. Zhao et al. (2007) used this approach to show the activation of RhoA by imposing a tension on integrins via collagen coated magnetic beads. Matthews (2006) used MTs to determine the effects of applying tension on magnetic beads coated with integrin ligands and showed the involvement of RhoA signaling pathways in the cellular response. Marjoram et al. (2016) evaluated single cell response to force pulses. They analyzed the effects of tension on VE-cadherin on endothelial cells, reporting an increase in RhoA activation and a decrease in Rac1 activation, a change in the phosphorylation levels of protein tyrosine, and a stiffening response to trains of short force pulses. Saphirstein et al. (2013) used MTs to investigate the mechanobiology of aortic tissue. Their results show that the focal adhesions of the vascular smooth muscle cells (VSMC), and particularly the FAK/Src complex, act as a regulator of aortic stiffness. Considering that the increase in aortic stiffness is linked to cardiovascular disease, the obtained results make FAs as a potential novel therapeutic target.

MTs have frequently been coupled with other techniques to gather more accurate information. Optical Magnetic Twisting Cytometry (OMTC), a technique of applying twisting torques to cells in culture, has been used by different groups (Fabry et al., 2001; Puig-de-Morales et al., 2004; Trepat et al., 2007) to study how different force regimes (i.e., modifying frequency or amplitude) applied to cells resulted in cellular reinforcement (stiffening) or fluidization (softening). Na and Wang (2008) combined FRET with MTs to study rapid mechanochemical signaling in live cells, and they demonstrated that pre-stressed cytoskeleton promoted rapid activation of Src protein upon force. With a similar setup, Poh et al. (2009) showed that a local stress in the physiologic magnitude range, applied through integrins on human airway smooth muscle cells, can directly and rapidly activate Rac GTPase, independently of Src activity. MTs have also been used in combination with microengineered platforms (Lin et al., 2012). The induced cellular contractile response to force and torque applied through nanowires bound to (or internalized by) bovine pulmonary artery smooth muscle cells (SMCs) was measured with arrays of elastic micropillars force sensors. The authors reported that the contractile response was connected to the actuation frequency, but was not dependent on the applied force or torque magnitude. Moreover, they observed a global enhancement of cell traction forces following the application of a localized torque. Recently, Bidan et al. (2018) applied a combination of MTs and TFM on deformable substrates enabling local and dynamic mechanical stimulation of cells plated on a continuous surface. Substrates consisted of a layer of soft elastomer embedding spatially arranged magnetic micropillars, that could be locally actuated by means of a MTs setup. The induced localized deformation of the substrate could be quantified by tracking fluorescent microbeads that were also embedded under the elastomer surface.

### Acoustic Tweezers (ATs)

In 1991, an article from Wu (1991) showed the possibility to stably trap a 270 μm latex particle or a cluster of frog eggs in a potential well generated by two collimated focused ultrasonic beams propagating along opposite directions in water. This technique, originally termed “acoustical tweezers”, is nowadays better known as ATs.

ATs are based on the concept that a stable potential well can be created by radiation pressure at the physical focal point of a focused ultrasonic beam (Wu and Du, 1990). This technique foresees the use of piezoelectric transducers positioned so that their beams focal points can be held a few millimeters apart, creating the potential well. Moving the transducers or tuning their frequency results in the displacement of the potential well and in the trapped objects to be moved alongside.

Manipulating biological specimens by acoustic waves, ATs present many advantages (Friend and Yeo, 2011; Ding et al., 2013) compared to similar techniques (i.e., optical and MTs). First of all, the emitted mechanical vibrations have such a low intensity (power intensity ca. 10 million times lower than OTs) that they have a minimal impact on cell viability and function, not altering cell characteristics. Moreover, particles or cells can be suspended into their preferred medium (i.e., culture medium or ECM) and moved in a contactless way, avoiding contamination. Also, there is no need for the manipulated cell to undergo surface modifications or labeling, so that cells maintain their shape, size, refractive index, charge and other native properties. Finally, the ATs platform can be made of a single, integrated micro-device, avoiding any moving parts and/or complicated setup procedures, which make the system very easy to use (Guo et al., 2016). In this regard, the implementation of surface acoustic wave (SAW) transducers (Ahmed et al., 2016) for on-chip manipulation of cells has been reported (Voiculescu and Nordin, 2012; Ding et al., 2014; Nguyen et al., 2017).

In summary, acoustic devices, not suffering from some of the drawbacks affecting other methods (i.e., no need of optical purified sample, possibility to manipulate large particles or cells, lower damage to biological samples) (Hwang et al., 2014; Huang et al., 2015; Li et al., 2015), have been demonstrated to successfully perform many microscale functions such as separation, alignment, patterning, enrichment and transportation of cells and microparticles (Friend and Yeo, 2011; Ding et al., 2013; Bourquin et al., 2014; Gesellchen et al., 2014; Guo et al., 2015; Li et al., 2015), and are considered to be a very attractive non-invasive approach for the manipulation of cells and particles for biomedical and biophysical applications (Lam et al., 2016).

An evolution of ATs with a high significance for the study of cell mechanobiology has been introduced by Fan et al. (2013). The authors used RGD-coated, ultrasound-excitable lipid microbubbles, that were bound to the membrane of living cells and targeted by ATs for their mechanical actuation. This technique, which goes under the name of acoustic tweezing cytometry (ATC), produces a rapid acoustic radiation force on the microbubbles that provokes the contractility of the intracellular cytoskeleton. Hence, ATC provides an effective method to apply mechanical stress to cells with no reported negative effects on cell physiology (Liu, 2016). As an example, Heureaux et al. (2014) engineered retinal pigment epithelial cells for the expression of bacterial mechanosensitive channel of large conductance (MscL), and challenged the cytoskeleton with localized stress by integrin-bound microbubbles, demonstrating the possibility to gate the MscL by a mechanical actuation that targets the integrin-focal adhesion-cytoskeleton connection. As a further improvement, Chen et al. (2015) developed an ATC methodology exploiting the acoustic interaction force between two cell-bound microbubbles, that is resulting from the scattering of the incident primary ultrasound field. The generated secondary acoustic radiation force (sARF) has the same magnitude for each of the microbubbles and is attractive, independently of the orientation of the primary ultrasound pulses. For this reason, two-bubble ATC (TB-ATC) technique provides advantages in the experimental setup and in the measurement of subcellular biomechanical properties. Using TB-ATC, the same research group demonstrated that ATC stimulation promotes cytoskeletal contractility and enhances osteogenesis of human mesenchymal stromal cells via YAP activation (Xue et al., 2017).

## Traction Force Microscopy (TFM)

Among passive approaches, TFM was one of the first and most broadly used techniques for measuring cell forces, providing maps of stresses at the cell surface. Ideally, every cell adherent to a soft substrate exerts a contractile force able to deform it to a measurable extent. Over the years, several TFM methods exploiting flexible 2D synthetic substrates of known mechanical properties have been reported. One of the first experiments introduced by Harris et al. (1980) used soft silicone rubber as cell substrate and provided maps of traction forces by measuring the size of the out-of-plane wrinkles generated by cell contraction. Although the force direction and magnitude are derived from wrinkles inspection by phase contrast microscopy, this approach is highly qualitative and the reported deformation cannot be compared across systems.

Therefore, researchers started to use fluorescence beads to monitor the deformation of thin hydrogel films, thereby gathering quantitative information. In standard TFM, micro/nanoscale fluorescent beads are embedded into the substrate or attached on the surface and used as fiduciary markers to be optically tracked in space and time. A common TFM experiment consists in imaging the bead positions in a stressed state (cell-loaded image) when cells seeded on the substrate start to contract. After releasing cell tractions by detaching the cell (i.e., by trypsinization), a new image is captured to determine the position of the beads in the unstressed state (unloaded or reference image). A displacement map of the deformed substrate – a map showing how each pixel deviates from its reference position due to the force exerted by the cell – is then derived from the two images either by single particle tracking or by digital image correlation (Franck et al., 2011).

To avoid the use of a reference image, recent advancements have involved the controlled dispensing of fluorescent markers (e.g., using the electrohydrodynamic NanoDrip printing technique) with regular spacing on the gel surface (Bergert et al., 2016). Alternatively, instead of using fluorescent beads distributed throughout the substrate, a modified version of TFM has been implemented by Balaban et al. (2001) who adapted soft lithography to embed patterns of fluorescent photoresist markers right under the substrate surface. Once the computational framework and imaging system are set up, measurements can be systematically performed and traction maps derived from the displacements through a variety of computational methods (Butler et al., 2002; Munoz, 2016). Since the beads are much smaller in size than a cell, TFM has allowed scientists to map forces with subcellular resolution enabling the characterization of the force dynamics involved in numerous biological and pathological processes, including cell adhesion, migration, differentiation, and metastatic potential (Engler et al., 2006; Indra et al., 2011; Jannat et al., 2011; Koch et al., 2012).

Polyacrylamide (PA) or silicon-based gels are typically used as substrates for TFM (Roca-Cusachs et al., 2017). Both types of gels exhibit a linear elastic behavior under deformations produced by cell traction, and their Young’s modulus (i.e., linear elastic modulus) can be varied over a range of several orders of magnitude. Furthermore, unlike native ECM, their mechanical properties do not change significantly during a single measurement since they are not degraded by biochemical factors released by the cells, including cell proteases. Although this is advantageous for cellular traction measurements, evidences from the literature revealed a significant effect of cell-mediated degradation of ECM matrices on cellular traction profiles (Khetan et al., 2013).

Thus, TFM has been rapidly extended from the computation of the 2D force field imparted by an individual cell on a 2D flat substrate to the quantification of forces in more realistic environments with a dramatic increment of computational time. The first step toward this goal has been the extension of TFM measurements to multicellular clusters (Trepat et al., 2009) and 2D substrates of arbitrary stiffness profiles (Sunyer et al., 2016). Furthermore, although the 2D TFM can approximate many experimental conditions, cells usually exert 3D forces on the adhering substrates and the normal traction component is often comparable to the in-plane one (Bastounis et al., 2014). Thus, to obtain a more accurate characterization of 3D traction field of cells cultured on a 2D substrate (often referred to as 2.5D tractions), TFM methods have been further modified and implemented to track bead displacements in 3D with confocal microscopy.

Another issue, even more tricky, is represented by the analysis of 3D force field exerted by cells encapsulated in 3D ECMs. In all the methods presented so far, cells are seeded on 2D substrates, while cells *in vivo* are embedded in 3D matrices, and their phenotype and shape is strikingly affected by the surrounding cell environment. However, greater use of 3D TFM is hindered not only by the limits in acquiring sub-micrometer scale features in 3D (Hall et al., 2013), but also by the more heterogeneous and mechanically complex properties of natively-derived fibrous components of the ECM compared to the synthetic materials used for 2D measurements (Hall et al., 2013). Unlike the 2D/2.5D cases, in which the properties of the substrate can be tightly engineered by researchers, the 3D ECM undergoes continuous remodeling that involves deposition, reorganization and degradation, precluding a straightforward interpretation of the deformation/force fields. Just to give an example, it is impossible to clearly discriminate if a large deformation in the proximity of a cell is induced by high cell traction or by a change in mechanical properties related to ECM remodeling. Moreover, natural ECMs are composed of fibers with highly non-linear behavior and randomly distributed in the same microscopic volume element. Although these 3D traction approaches have still limited applicability compared to 2D TFM, they have already evidenced a different cell mechanical behavior between 3D and 2D environments, as demonstrated for forces applied by MDA-MB-231 breast carcinoma cells in 3D polymers that appear to be independent of concentration and stiffness of the surrounding matrix (Steinwachs et al., 2016).

Collagen type I hydrogel is a common ECM-mimicking material for 3D cell culture. Particle tracking techniques have successfully led to the quantification of pericellular collagen deformations during tumor cell invasion and migration through a 3D collagen matrix (Bloom et al., 2008; Koch et al., 2012). However, the non-linear force-displacement response of this hydrogel prevents quantification of traction forces from the deformations using classical mechanics approaches. Other studies report quantitative finite-element based methods to measure cell-generated forces in physiologically-mimicking 3D biopolymeric matrices with highly non-linear mechanical response such as collagen and fibrin gels (Steinwachs et al., 2016). Alternatively, to avoid the issues associated with non-linearity, 3D traction fields have been computed by Legant et al. (2010) using synthetic, matrix metalloprotease (MMP)-cleavable polyethylene glycol (PEG) gels. These synthetic hydrogels exhibit linearly elastic behavior in the range of deformations produced by single cells, and the possibility to track beads in this material has successfully led to the measurement of cellular tractions in 3D. Although a significant improvement has been made, this method still assumes that the material only undergoes elastic deformation (Palacio et al., 2013) and a lot of work still needs to be done to fully incorporate non-linear and poroelastic models of hydrogel substrates into routine algorithms improving data quality at large deformations.

Since traction forces play a fundamental role in many biological processes including embryogenesis, angiogenesis, inflammation, wound healing and metastasis, the application of TFM has allowed better understanding of cellular and molecular mechanisms of these processes (Wang and Li, 2010; Li and Wang, 2011; Malandrino et al., 2018).

One of the basic TFM biological applications is the measurement of traction forces imparted by single cells on a 2D substrate. For example, a maximum displacement of around 1.2 μm, corresponding to a traction stress of about 250 Pa, has been found by Yang et al. (2006) for human patellar tendon fibroblasts seeded on a type I collagen-coated PA substrate. More interestingly, as traction forces vary depending on cell type, TFM measurements can be useful in detecting cell phenotypic changes. On this basis, TFM has been successfully applied to detect differentiated cells and to distinguish between fibroblasts and myofibroblasts obtained from the differentiation of rabbit corneal stromal cells in conditioned media (Chen et al., 2007). Going beyond the measurement of traction forces applied by single cells, TFM has also found applications to explore the behavior of cellular aggregates (Li et al., 2009), with particular regard to the mechanisms behind collective cell migration (Trepat et al., 2009).

In addition, taking advantage of microfluidics and micro-technologies, a recent trend is represented by the integration of TFM with miniaturized mechanically actuated systems to investigate cellular forces under dynamic conditions mimicking physiological stimuli, e.g., shear flow (Shiu et al., 2004), mechanical stretch (Gavara et al., 2008), and chemokine gradients (Del Alamo et al., 2007).

In this way, TFM has been applied to observe spatiotemporal patterns of forces in settings that are more representative of physiological and pathological *in vivo* conditions (Cho et al., 2016). As a major outcome, this technique could have a pivotal role in the development of *in vitro* cell culture models of several diseases, especially if associated with changes in cell contractility (e.g., hypertension, muscle dystrophy, etc.), to ease diagnostic screening and therapeutic treatments. A leading example is represented by the *in vitro* model developed by Li et al. (2008) who investigated the contractility of micropatterned C2C12 skeletal muscle cells using TFM with the aim to obtain a fast screening platform for therapy of Duchenne muscular dystrophy.

While effective in a variety of biological applications, further advancements in TFM are needed to improve spatial resolution, enable real time assays, and measure forces within 3D matrices in a high-throughput manner (Colin-York and Fritzsche, 2018).

## Microengineered Platforms

As an alternative to TFM, microfabricated platforms have been investigated to measure cellular tractions in controlled mechanical environments. A variety of approaches has been described in the literature, but a classification into two major classes can be performed (Rajagopalan and Saif, 2011); hard silicon-based devices and soft polymer/gel devices.

The first category includes silicon devices fabricated through integrated circuit manufacturing processes, the so-called Micro-Electro-Mechanical Systems (MEMS). In this case, cells are contacted with compliant silicon elements that deform in response to cellular forces altering their electrical response, as extensively reviewed by Polacheck and Chen (2016). The design flexibility of MEMS translates into the possibility of force measurements along multiple axes, coupled to unprecedented measurable force range (10^−12^ ÷ 10^−3^ N; Sun and Nelson, 2007). Notable examples of MEMS devices for cell biomechanical characterization are represented by the work of Matsudaira et al. (2017) who developed a silicon piezoresistive cantilever platform for measuring the beating contraction force of iPS-derived cardiomyocytes, and that of Takahashi et al., 2016 who designed a MEMS force plate to measure single cell horizontal and vertical traction forces. Although the majority of these devices are passive, approaches to single-cell mechanical actuation using MEMS have to be acknowledged (Scuor et al., 2006; Antoniolli et al., 2014), also integrating force sensing capabilities besides actuation ones (Fior et al., 2011; Zhang and Dong, 2012).

The second category of microengineered platforms encompasses soft polymer and gel microsystems obtained through soft-lithography techniques. On the one hand, these polymer-based devices require the use of image analysis to quantify cell-induced displacements due to the difficulties in the integration of electronic components into such devices. On the other hand, the higher biocompatibility and optical transparency of these systems, together with the possibility to easily tune surface chemistry and mechanical properties to better mimic mechanical *in vivo* environment, have made them increasingly popular for mechanobiology studies (Rajagopalan and Saif, 2011).

Among polymer-based substrates, microfabricated (vertically arranged) micropillar arrays have been applied to the measurement of forces exerted by single adhesion sites of a cell in constructs with as few as 100–600 cells (Polacheck and Chen, 2016). These structures are obtained by soft lithography, consisting in replica molding of a patterned silicon master using commercial silicone elastomer (PDMS). After microcontact printing of ECM proteins over the micropillar tips, cells can adhere and spread, exerting contractile forces that deflect the underlying pillars as simple cantilever beams. If the deflection is sufficiently small compared with the height of the posts, the displacement of the cantilever beam tip and the force are proportional. Thus, traction forces can be directly calculated from optically measured micropillar deflections, provided that the constant of the spring is known. An exhaustive description of how to fabricate the silicon masters and elastomeric replicas, as well as cell culture procedures, immunofluorescence imaging and traction force evaluation on these substrates can be found in previous works (Yang et al., 2011; Gupta et al., 2015).

PDMS micro/nanopillars have been successfully applied to investigate forces exerted by different cells, including fibroblasts, endothelial cells, stem cells, etc., at both cellular and subcellular levels (Nelson et al., 2005; Fu et al., 2010; Ghassemi et al., 2012). In particular, these systems have gained great interest as tool for measuring forces in cells that cannot be isolated or easily expanded, such as cardiomyocytes and human iPSCs, leading to high-throughput, low volume screening platforms for cardiac studies (Boudou et al., 2012; Serrao et al., 2012; Hinson et al., 2015).

As for TFM, micropillar arrays have also been integrated with miniaturized actuating systems to detect cell–ECM interactions under dynamic conditions mimicking physiological ones (Shao and Fu, 2014). For instance, coupling micropillar arrays to microfluidic channels allowed measurement of cell contractile forces under laminar shear flow (Lam et al., 2012a) or during chemotaxis-regulated cell migration (Ricart et al., 2011). Mechanical actuation of micropillar arrays was also pursued as a tool to study the response of cells to external mechanical stimuli, exploiting either vacuum-driven mechanical stretching (Lam et al., 2012b; Mann et al., 2012) or magnetic actuation of micropillars embedding magnetic nanowires (Sniadecki et al., 2007).

Compared to TFM measurements, the use of micro/nanopillar sensing elements has a few advantages. First, a reference image is not required since displacements can be calculated from undeformed pillar positions. Second, cellular forces are derived from the deformation of single polymeric structures, leading to a simpler and less computationally expensive calculation. Finally, heterogeneous mechanical environments can be obtained by simply altering micropillar geometries that influence substrate stiffness.

Stiffness can be varied over a wide range of values through the modulation of pillar structural parameters, such as height and diameter. Tan et al. (2003) constructed circular posts with 3 μm diameter, 11 μm height and 6 μm spacing, corresponding to a stiffness of 32 nN⋅μm^−1^ per post using standard photolithography and PDMS replica molding. Access to improved microfabrication processes such as high-resolution lithography and deep reactive-ion etching (DRIE) has led to the reduction of pillar diameter to the sub-micrometer level, and to the increase of micropillar aspect ratio (du Roure et al., 2005; Yang et al., 2007; Kim et al., 2009). Using a combination of nanosphere lithography and plasma etching, Shiu et al. (2018) have recently further scaled down micropillar geometries, obtaining epoxy-based photoresist structures that were 250 nm in diameter and 1.5 μm in height, with a spacing of 800 nm and a stiffness of 79 nN⋅μm^−1^.

It is worth mentioning that the discrete adhesive surface that cells sense on micropillars might affect the recruitment of integrins and adhesion proteins. This may in turn influence the morphology of cell–ECM adhesions, thereby creating a bias in the measurements. In this regard, the use of sub-micrometer pillars with reduced center-to-center distance (Yang et al., 2007; Fu et al., 2010; Ghassemi et al., 2012) might provide a closer resemblance to continuous tissue culture substrates as demonstrated by the increasing number of papers reporting comparable behaviors for several cell types (e.g., fibroblasts, endothelial cells, smooth muscle cells, MSCs, and embryonic stem cells) when cultured on PDMS micro/nano-pillar arrays and on continuous substrates (Yang et al., 2007, 2011). It also has to be acknowledged that micropillar technology restricts the achievable stiffness range compared to continuum substrates used in TFM (Miroshnikova et al., 2018), not achieving the fabrication of ultra-compliant arrays equivalent to the softest PA gels. Moreover, although the elastomeric replicas can be manufactured in a standard laboratory, the production of the original master requires sophisticated microfabrication facilities and equipment that are not common in research laboratories. Additionally, calculating an effective stiffness of micropillar substrates to be compared with physiological parameters is not an easy task, even if some approaches have been proposed (Ghibaudo et al., 2008). The simplest and most common approach is to approximate the polymeric material as linear elastic, and to express the spring constant of the micropillar from the classical beam theory^1^. However, such a theory is only valid in the linear regime of small deformations (Li et al., 2007). Xiang and LaVan (2007) have integrated shear strain and large deflection theory and demonstrated that, for deflection-to-length ratios less than 20%, the difference between using the small or the large deflection model was less than 10%. Furthermore, other issues related to the non-negligible effect of the elastic deformation of the pillar substrate (Zhao et al., 2005) and to the effect of materials viscoelasticity under dynamic analysis with different loading time/frequency (Lin et al., 2008) should be taken into account. A detailed survey of the advantages and disadvantages of the different analytical and computational models is out of the scope of this review and has been exhaustively described elsewhere (Zheng and Zhang, 2011).

## Conclusion and Future Outlook

Cellular mechanics is of primary importance in many pathophysiological processes. Mechanical forces elicit several biological processes in a cell, not only changing the ability of a cell to respond to exogenous signals and stimuli, but also dramatically influencing the way in which differentiation decisions are made during development.

The classical methods of fluorescence microscopy and spectroscopy can be used to detect the position, distribution and dynamics in real-time of single molecules, but they are not able to provide detailed information on the mechanical features, on the functional state of biomolecules or on the interactions between biological systems on a molecular scale. In the last decades, remarkable advancements have been made in devising novel and effective techniques for identifying and handling single molecules for the structural and functional study of biomolecules in physiological conditions. These techniques can be divided into active and passive, and they allow studying *in vitro* the mechanical properties of cells by generating or detecting forces down to the pN range.

In this review, various methods applied to the field of mechanobiology have been described. Attention was focused on active measurement methods (i.e., atomic force microscopy, optical/magnetic/acoustic tweezing) and on passive ones (TFM, MEMS, and microfabricated substrates). This review highlights the improvements brought to the single techniques to better characterize biological entities. Among these advancements, we emphasize the importance of investigating the biological response in conditions similar to the physiological environment and for longer time without inducing cell damage, along with the study of cellular responses to a given biomechanical stimulus in 3D conditions, thus recreating the tissue microenvironment, in order to obtain a response closer to reality. The proper measurement of physico-mechanical entities, on a cellular and subcellular scale, in a physiological or pathological condition is indeed extremely challenging due to the complexity of recapitulating an *in vivo* context that contributes to the generation and propagation of cellular forces. Discriminating the contribution of each component in the complex *in vivo* microenvironment of a living tissue is not completely feasible yet. Except for the most recent advances in the use of high-resolution OTs (that suffer from limitations in analyzing events located deep in tissues) and MTs combined with single molecule confocal microscopy and FRET technology to measure fluid forces *in vivo* and to track mechanotransduction in living cells, a single cell must be isolated from its surrounding environment and cultured on a suitable substrate in order to allow researchers to perform measurements and analyze its mechanical behavior, which is a much more simplified context compared to native tissues. Furthermore, there are many physico-chemical conditions (e.g., temperature, surface energy) and mechano-structural factors (e.g., strain stiffening, pressure, cell geometry) that may have significant effects on measurement accuracy. However, these conditions cannot be easily recreated *in vitro*. Therefore, to date, such aspects represent a significant limitation to the correct prediction of cell mechanical behaviors.

Among the different methodologies taken into consideration in mechanobiology studies, AFM represents a well-established and widely used technique, which can give a topographic image of cells and biomolecules, enabling a deeper investigation into the dynamic properties of the analyzed sample, for instance in terms of molecular interactions required for the adhesion of a single cell to a substrate. Furthermore, technical improvements to the setup have allowed AFM measurements to be performed in culture conditions and in combination with fluorescence microscopy. However, the technique has limitations in terms of measurement speed and costs of the instrument itself.

Unlike AFM, tweezing technologies allow users to study both cellular stiffness and intracellular mechanisms (such as endocytosis) using specific molecules or marked particles. Tweezing techniques still suffer from some limitations: in particular, OTs can damage cells after prolonged exposure to high powered lasers. This problem has been partially overcome by implementing MTs and ATs.

On the other hand, MEMS technology affords the possibility to fabricate extremely complex electromechanical systems and platforms either in 2D or 3D fashion, at the micrometer scale. By using a wide range of materials having different chemical or physical features, MEMS devices can integrate miniaturized mechanical and electro-mechanical elements that can be exploited for diverse purposes.

Thanks to major steps forward in the development of microfabrication and microfluidics, cell biomechanics and mechanotransduction mechanisms can be studied in dynamic conditions, simulating pathophysiological stimuli, in a 3D-like microenvironment. These fast-growing technologies have been advancing our understanding on how various cell types and tissues sense and respond to mechanical stimuli, opening the way toward new strategies for investigating dynamic and complex biological phenomena, such as embryonic development and migratory response of tumor cells. A correct quantification and understanding of cell mechanical behavior may indeed provide an essential foundation for studying and envisaging the development of several diseases.

In addition, TFM methods have been combined with 3D systems, with the support of confocal microscopy, for observing tractions on cell surface, and for mapping 3D stress and strain fields of single cells encapsulated in elastic and viscoelastic materials. The future of this technology may be represented by its application to synthetic and natural fibrous gels for monitoring traction fields of isolated cells embedded in either synthetic or naturally derived fibrous materials, such as collagen, with the ambitious aim of recapitulating a physiologically relevant environment in terms of biophysical and biochemical parameters.

In conclusion, countless techniques have been developed for the study of mechanobiology and the understanding of the role of mechanical stimuli on cellular response. Altogether, the coupling of biological, physico-chemical, and engineering knowledge has allowed scientists to develop technological platforms that hold great promise for a comprehensive study of cell mechanobiology.

As a future perspective, we expect technological progresses to drive the advancement of the above described methods, resulting in an extended range of applicable and measurable forces, and an improved spatio-temporal resolution. The next generation of tools to measure cellular forces is expected to create complex cell microenvironments through the use of combinatorial guidance cues in a single experiment, so that single cells can experience a dynamically changing set of mechanical and biochemical conditions more representative of *in vivo* settings. In this regard, we will probably assist to a tighter convergence of the technologies, as expected for high-speed AFM and OTs (Ando, 2018) in order to combine nanoscale resolution imaging with manipulation capabilities at the molecular level. The attention of researchers in the field of mechanobiology will reliably be drawn to the measurement of cell and tissue mechanical properties *in vivo.* In this scenario, MEMS devices have the potential to detain a leading role in light of their extremely wide force range and design flexibility.

The challenge remains in translating the accumulating evidence of mechanical regulation of cell functions in physiology and disease into next-generation diagnoses and treatments.

## Author Contributions

FB, SG, MG, PM, AB, MT, and AR wrote and revised the manuscript.

## Conflict of Interest Statement

The authors declare that the research was conducted in the absence of any commercial or financial relationships that could be construed as a potential conflict of interest.

## Footnotes

1 Under small deflection assumptions, pillar elastic constant can be expressed as k = 3π ED^4^/64H^3^, where E, D and H are the elastic modulus of PDMS, the diameter and the height of the pillar, respectively.
